# Underweight patients experience higher inpatient complication and mortality rates following acetabular fracture

**DOI:** 10.1007/s00590-023-03739-z

**Published:** 2023-09-29

**Authors:** Julian Wier, Reza Firoozabadi, Andrew Duong, Joseph T. Patterson

**Affiliations:** 1 Department of Orthopaedic Surgery, Keck School of Medicine of the University of Southern California, 1520 San Pablo Street, Suite 2000, Los Angeles, CA 90033-5322 USA; 2 Department of Orthopedics and Sports Medicine, University of Washington, Seattle, WA USA

**Keywords:** Pelvis, Acetabulum, Fracture, Underweight, Body mass index, Outcomes, Complications, Older adults

## Abstract

**Purpose:**

Underweight patients experience poor outcomes after elective orthopaedic procedures. The effect of underweight body mass index (BMI) on complications after acetabular fracture is not well-described. We evaluate if underweight status is associated with inpatient complications after acetabular fractures.

**Methods:**

Adult patients (≥ 18 years) presenting with acetabular fracture between 2015 and 2019 were identified from Trauma Quality Program data. Adjusted odds (aOR) of any inpatient complication or mortality were compared between patients with underweight BMI (< 18.5 kg/m^2^) and normal BMI (18.5–25 kg/m^2^) using multivariable logistic regression and stratifying by age ≥ 65 years.

**Results:**

The 1299 underweight patients aged ≥ 65 years compared to 11,629 normal weight patients experienced a 1.2-times and 2.7-times greater aOR of any complication (38.6% vs. 36.6%, *p* = 0.010) and inpatient mortality (7.9% vs. 4.2%, *p* < 0.001), respectively. The 1688 underweight patients aged 18–64 years compared to 24,762 normal weight patients experienced a 1.2-times and 1.5-times greater aOR of any inpatient complication (38.9% vs. 34.8%, aOR *p* = 0.006) and inpatient mortality (4.1% vs. 2.5%, *p* < 0.001), respectively.

**Conclusion:**

Underweight adult patients with acetabular fracture are at increased risk for inpatient complications and mortality, particularly those ≥ 65 years old.

**Level of Evidence:**

Prognostic Level III.

**Supplementary Information:**

The online version contains supplementary material available at 10.1007/s00590-023-03739-z.

## Introduction

Acetabular fractures occur with a reported incidence of 5–11 fractures per 100,000 person-years. [1, 2] Patient age, frailty, and comorbidity burden are associated with adverse outcomes. [3, 4] Low energy acetabular fractures in older adults are increasing in incidence faster than other fragility fractures. [1] These older patients face serious risks of adverse outcomes including 18–22% mortality and 25–68% non-fatal adverse events, similar to older adults with hip fracture. [3, 4]. Acetabular fractures occur in both young and elderly populations, with a reported incidence of 5 to 11 fractures per 100,000 person-years [1, 2]. While historical reports have focused on high energy trauma in adults, the low impact geriatric acetabular fractures are increasing in their incidence and can be used to delineate two distinct patient populations [1, 5]. Mortality and non-fatal complications [3, 4], patient age, frailty remain non-trivial after acetabular fracture and are likely influenced by patient age and comorbidity burden; however, more data are required to optimize risk stratification and are associated with adverse outcomes [3, 4, 6]. Low energy acetabular fractures in older adults are increasing in incidence faster than other fragility fractures [1, 5]. These older patients face serious risks of adverse outcomes including 18–22% mortality and 25–68% non-fatal adverse events, similar to older adults with hip fracture [3, 4].

Less than 10% of the global adult population is underweight (body mass index [BMI] < 18.5 kg/m^2^) [7]. Underweight patients experience approximately 50% greater risk of mortality of adverse events following elective orthopaedic procedures and hip fracture surgery [8–10]. However, little is known about the influence of being underweight on patient outcomes following acetabular fracture surgery, particularly in younger and middle-aged adults [11]. Previous studies aimed at determining the effects of BMI on complications after acetabular fractures have largely focused on obesity and older adults [11].

We sought to evaluate whether underweight adults with acetabular fracture experienced different rates of adverse events compared with normal weight adults. We hypothesized that underweight status would be associated with increased risk of mortality and any inpatient complication, adjusting for patient factors and stratifying between younger and older adults (age ≥ 65 years) [12].

## Patients and methods

The Trauma Quality Improvement Program (TQIP) data from the American College of Surgeons were retrospectively queried for adult patients (≥ 18 years) who presented to a participating trauma center between 2015 and 2019 with a new diagnosis of acetabular fracture. TQIP is a deidentified database of injured patients presenting to over 875 North American trauma centers Abbreviated Injury Scale (AIS 2005) codes (Supplementary Table S1) that were used to identify acetabular fractures using the National Trauma Data Standard (NTDS) criteria [13]. International Statistical Classification of Diseases and Related Health Problems 10th coding, Procedural Coding System (ICD-10-PCS) codes (Supplementary Table S1) identified the use of open reduction internal fixation (ORIF) or percutaneous/closed reduction internal fixation (CRIF) for the acetabular fracture. Patients were excluded if declared dead on arrival, had disseminated cancer, or were receiving chemotherapy. Covariates with > 20% missing data for primary study outcomes or model covariables were excluded. Older adults were identified by age ≥ 65 years old as previously classified by Ly and Swiontkowski. [12] Patients with normal BMI (18.5–25 kg/m^2^) were then compared to underweight patients (< 18.5 kg/m^2^).

The primary outcomes were any inpatient complication, defined as at least one instance of acute kidney injury (AKI), cardiac arrest, central line associated blood stream infection (CLABSI), catheter associated urinary tract infection (CAUTI), deep surgical site infection (SSI), deep vein thrombosis (DVT), intubation, osteomyelitis, pulmonary embolism (PE), pressure ulcer, respiratory failure, sepsis, stroke, superficial SSI, unplanned admission to the intensive care unit (ICU), unplanned return to the operating room (OR), or ventilation associated pneumonia (VAP) and inpatient mortality. Secondary outcomes included rates of acute kidney injury, cardiac complication (MI or cardiac arrest), inpatient mortality, infectious complications (CLABSI, CAUTI, SII, sepsis, or VAP), pulmonary complication (respiratory failure, unplanned intubation, or VAP), and venous thromboembolism (VTE).

Bivariable regressions on the associations between study outcomes and BMI class were conducted. Subsequently, multivariable regressions were conducted to account for potential confounding factors using demographic, comorbidity, injury severity, admitting facility, and intervention data. (Supplementary Table S2) The final models were selected via stepwise minimization of the Akaike’s Information Criterion with subsequent minimization of the Bayesian Information Criterion. Multicollinearity was assessed via evaluation of variance inflation factors (VIF). Covariates with VIF > 10 were excluded from the model in a stepwise fashion. These analyses were repeated for all study outcomes. All statistical analyses were performed using Stata Version 17.0 (College, Station, TX), reporting 2-sided p values with the level of significance for *p* < 0.050.

## Results

**Fig. 1:**
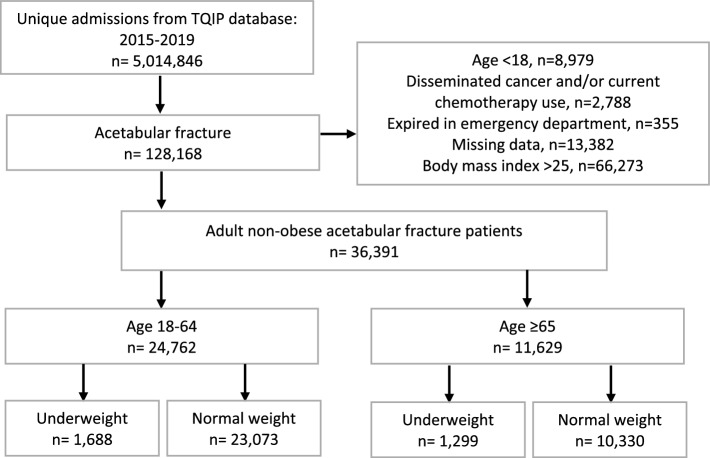
STROBE diagram of patient selection

A total of 36,391 patients with acetabular fracture were available for analysis; of these, 11,629 (32.0%) were ≥ 65 years old and 24,762 (68.0%) were 18–64 years old. Underweight patients represented a minority for both older (1299 [11.1%]) and younger adults (1688 [6.8%]). (Fig. 1). The mean BMI of underweight patients in both age cohorts was 17.0 kg/m^2^ (95%-confidence interval [CI] = 16.9–17.1), while the mean BMIs for the normal weight cohorts were 22.3 kg/m^2^ (95%-CI = 22.3–22.3) and 22.5 kg/m^2^ (95%-CI = 22.5–22.5) in the older and younger cohorts, respectively.

### Demographics, injury characteristics, and treatments in older patients

Underweight older adults were 1.5 years older than normal weight patients (79.2 vs. 77.7 years, *p* < 0.001) and more frequently female (Table 1). Underweight patients carried significantly more burden of six of the 18 comorbid medical conditions evaluated (range 0.2–7.5% absolute difference), with notably greater rates of COPD (18.1% vs. 10.6%, *p* < 0.001) and functionally dependent status (17.6% vs. 12.7%, *p* < 0.001; Table 1). On admission, the underweight cohort was less severely injured; however, rates of concomitant injury were similar (Table 1). Underweight patients were more commonly admitted to non-teaching level II or III trauma centers (Supplementary Table S3). The majority of acetabular fractures involved one column in both cohorts (44.9% vs. 44.8%; Table 1). Underweight patients less frequently received operative fixation of their acetabular fracture than normal weight patients (8.2% vs. 13.3%, *p* < 0.001; Table 1). Of those who required operative treatment, underweight patients were more commonly treated within one to five days of admission than the normal weight cohort (74.5% vs. 68.1%, *p* < 0.001; Table 1). No significant differences in length of hospital stay were observed (7.3 days [95%-CI = 7.2–7.4] vs. 7.1 days [95%-CI = 6.7–7.6], *p* = 0.450); however, underweight patients more frequently underwent unplanned intubation during their hospital admission than normal weight patients (11.4% vs. 8.9%, *p* = 0.004; Table 1).

**Table 1:** Demographic, comorbidity, admission injury severity, and intervention characteristics

Patients aged ≥ 65 years	Underweight *N* = 1299 (%)	Normal weight *N* = 10,330 (%)	*P* value
*Demographics*			
Mean age, years	79.2 (95% CI 78.8–79.6)	77.7 (95% CI 77.5–77.8)	**< 0.001**
Sex, male	419 (32.3%)	5,294 (51.2%)	**< 0.001**
BMI (kg/m^2^)	17.0 (95% CI 16.9–17.1)	22.3 (95% CI 22.3–22.3)	**< 0.001**
*Race*			
American Indian	2 (0.2%)	34 (0.3%)	0.371
Asian	32 (2.5%)	253 (2.4%)	
Black	67 (5.2%)	472 (4.6%)	
Hispanic	95 (7.3%)	850 (8.2%)	
Pacific islander	2 (0.2%)	11 (0.1%)	
Other	1 (0.1%)	1 (0.0%)	
White	1100 (84.7%)	8709 (84.3%)	
*Comorbidities*			
Alcoholism	47 (3.6%)	449 (4.3%)	0.221
Anticoagulant use	86 (6.6%)	829 (3.6%)	0.076
Bleeding disorder	98 (7.5%)	658 (6.4%)	0.216
CHF	99 (7.6%)	716 (6.9%)	0.359
Cirrhosis	21 (1.6%)	201 (1.9%)	0.414
COPD	235 (18.1%)	1090 (10.6%)	**< 0.001**
CVA history	76 (5.9%)	533 (5.2%)	0.292
Dementia	181 (13.9%)	1182 (11.4%)	**0.009**
Diabetes	163 (12.5%)	1619 (15.7%)	**0.003**
ESRD	46 (3.5%)	394 (3.8%)	0.627
Functional dependence	228 (17.6%)	1310 (12.7%)	**< 0.001**
Hypertension	590 (45.4%)	4545 (44.0%)	0.331
Mental disorder	139 (10.7%)	958 (9.3%)	0.097
Myocardial infarction history	26 (2.0%)	224 (2.2%)	0.696
PAD	40 (3.1%)	202 (2.0%)	**0.007**
Smoking	165 (12.7%)	997 (9.7%)	**0.001**
Steroid use	43 (3.3%)	232 (2.2%)	**0.017**
Substance abuse	32 (2.5%)	216 (2.1%)	0.381
*Admission injury characteristics*			
GCS	14.6 (95% CI 14.6–14.7)	14.6 (95% CI 14.6–14.6)	0.378
ISS	8.7 (95% CI 8.3–7.2)	9.4 (95% CI 9.3–9.6)	**0.004**
Abbreviated injury severity: lower extremity	2.3 (95% CI 2.2–2.3)	2.2 (95% CI 2.2–2.3)	0.925
*Acetabular fracture pattern*			
One column	582 (44.8%)	4641 (44.9%)	0.078
Transverse	83 (6.4%)	757 (7.3%)	
Associated both column	153 (11.8%)	1495 (14.5%)	
Other	481 (37.0%)	3437 (33.3%)	
Open fracture	63 (4.8%)	426 (4.1%)	0.219
Femur fracture	113 (8.7%)	858 (8.3%)	0.629
Tibia fracture	34 (2.6%)	374 (3.6%)	0.064
Unstable pelvic ring disruption	108 (8.3%)	956 (9.3%)	0.129
*Interventions*			
Definitive acetabular fracture fixation			
Non-operative	1193 (91.8%)	8953 (86.7%)	**< 0.001**
CRIF	14 (1.1%)	128 (1.2%)	
ORIF	92 (7.1%)	1,249 (12.1%)	
Time to acetabular fixation			
< 24 Hours	19 (17.9%)	271 (19.7%)	**< 0.001**
1–3 Days	54 (50.9%)	646 (46.9%)	
3.1–5 Days	25 (23.6%)	292 (21.2%)	
> 5 Days	10 (9.4%)	168 (12.2%)	
Units of pRBCs given within 24 h of admission	56 (4.3%)	562 (5.4%)	0.087
Total length of stay, days	7.1 (95% CI 6.7–7.6)	7.3 (95% CI 7.2–7.4)	0.450
ICU admission	341.0 (26.3%)	2749 (26.6%)	0.781
ICU length of stay, days	6.0 (95% CI 5.2–6.7)	6.0 (95% CI 5.8–6.3)	0.905
Intubation	148.0 (11.4%)	923.0 (8.9%)	**0.004**
Ventilator days	7.6 (95% CI 6.10–9.0)	7.3 (95% CI 6.8–7.9)	0.744

Patients aged 18–64 years	Underweight *N* = 1688 (%)	Normal weight *N* = 23,074 (%)	*P* value
*Demographics*			
Mean age, years	37.6 (95% CI 36.9–38.3)	37.8 (95% CI 37.6–38.0)	0.519
Sex, male	940 (55.7%)	15,903 (68.9%)	**< 0.001**
BMI (kg/m^2^)	17.0 (95% CI 16.9–17.1)	22.5 (95% CI 22.5–22.5)	**< 0.001**
*Race*			
American Indian	14 (0.8%)	162 (0.7%)	**< 0.001**
Asian	74 (4.4%)	591 (2.6%)	
Black	275 (16.3%)	4,093 (17.7%)	
Hispanic	239 (14.2%)	3,396 (14.7%)	
Pacific islander	3 (0.2%)	66 (0.3%)	
Other	0 (0.0%)	5 (0.0%)	
White	1083 (64.2%)	14,761 (64.0%)	
*Comorbidities*			
Alcoholism	129 (7.6%)	1749 (7.6%)	0.926
Anticoagulant use	29 (1.7%)	342 (1.5%)	0.441
Bleeding disorder	46 (2.7%)	324 (1.4%)	**< 0.001**
CHF	30 (1.8%)	307 (1.3%)	0.126
Cirrhosis	30 (1.8%)	332 (1.4%)	0.263
COPD	103 (6.1%)	792 (3.4%)	**< 0.001**
CVA History	26 (1.5%)	323 (1.4%)	**< 0.001**
Dementia	21 (1.2%)	255 (1.1%)	0.627
Diabetes	112 (6.6%)	1367 (5.9%)	0.600
ESRD	35 (2.1%)	278 (1.2%)	0.234
Functional dependence	48 (2.8%)	393 (1.7%)	**0.002**
Hypertension	167 (9.9%)	2001 (8.7%)	**0.001**
Mental disorder	171 (10.1%)	2007 (8.7%)	0.087
Myocardial infarction history	17 (1.0%)	239 (1.0%)	0.910
PAD	19 (1.1%)	248 (1.1%)	0.845
Smoking	516 (30.6%)	6729 (29.2%)	0.220
Steroid use	27 (1.6%)	286 (1.2%)	0.201
Substance abuse	188 (11.1%)	2784 (12.1%)	0.257
*Admission injury characteristics*			
GCS	13.7 (95% CI 13.5–13.8)	13.7 (95% CI 13.7–13.8)	0.267
ISS	15.2 (95% CI 14.6–15.7)	15.3 (95% CI 15.2–15.5)	0.619
Abbreviated injury severity: lower extremity	2.5 (95% CI 2.4–2.5)	2.5 (95% CI 2.5–2.5)	0.565
Acetabular fracture type			
One column	830 (49.2%)	11,329 (49.1%)	0.295
Transverse	142 (8.4%)	2162 (9.4%)	
Associated both column	209 (12.4%)	2670 (11.6%)	
Other	507 (30.0%)	6913 (30.0%)	
Open	93 (5.5%)	1078 (4.7%)	0.118
Femur fracture	272 (16.1%)	4132 (17.9%)	0.063
Tibia fracture	192 (11.4%)	2647 (11.5%)	0.904
Unstable pelvic ring disruption	292 (17.3%)	3559 (15.4%)	**0.040**
*Interventions*			
Definitive acetabular fracture fixation			
Non-operative	1339 (79.3%)	17,102 (74.1%)	**< 0.001**
CRIF	22 (1.3%)	438 (1.9%)	
ORIF	327 (19.4%)	5534 (24.0%)	
Time to acetabular fixation			
< 24 Hours	66 (18.9%)	1395 (23.4%)	**< 0.001**
1–3 Days	153 (43.8%)	2629 (44.0%)	
3.1–5 Days	72 (20.6%)	1123 (18.8%)	
> 5 Days	58 (16.6%)	825 (13.8%)	
Units of pRBCs given within 24 h of admission	171 (10.1%)	2729 (11.8%)	**0.036**
Total length of stay, days	10.0 (95% CI 9.5–10.7)	10.0 (95% CI 9.9–10.2)	0.847
ICU admission	688 (40.8%)	9,258 (40.1%)	0.607
ICU length of stay, days	7.2 (95% CI 6.5–7.8)	7.2 (95% CI 7.0–7.4)	0.989
Intubation	382 (22.6%)	4,742 (20.6%)	**0.042**
Ventilator days	7.2 (95% CI 6.4–8.1)	7.3 (95% CI7.1–7.6)	0.874

*p*-values in bold indicate statistical signficance at < 0.05

*BMI* body mass index, *CHF* congestive heart failure, *COPD* chronic obstructive pulmonary disease, *CRIF* closed reduction internal fixation, *CVA* cerebrovascular accident, *ESRD* end stage renal failure, *GCS* glasgow coma score, *ICU* intensive care unit, *ISS* injury severity score, *ORIF* open reduction internal fixation, *PAD* peripheral arterial disease, *pRBCs* packed red blood cells

### Outcomes in older patients

Significantly higher rates of inpatient complications mortality (7.9% vs. 4.2%, *p* < 0.001) and cardiac complications (5.9% vs. 3.2%, *p* < 0.001) were observed in the underweight cohort on bivariate analysis (Table 2). After adjusting for confounding covariates including age, underweight status was associated with 1.2 times greater adjusted odds of any complication (95%-CI = 1.0–1.4, *p* = 0.010), 2.7 times greater adjusted odds of inpatient mortality (95%-CI = 2.0–3.6, *p* < 0.001), 2.1 times greater adjusted odds of cardiac complications (95%-CI = 1.6–2.7, *p* < 0.001), and 1.4 times greater adjusted odds of pulmonary complications (95%-CI = 1.1–1.8, *p* < 0.001). Infectious complications, AKI, and VTE were not significantly different between the two groups after multivariable regression (all *p* > 0.05) (Table 2).

**Table 2:** Bivariable and multivariable logistic regression analyses on odds of inpatient complications

Patients aged ≥ 65 years	Underweight *N* = 1299	Normal weight *N* = 10,330	Bivariable logistic regression		Multivariable logistic regression	
Complication	*N* (%)	*N* (%)	OR(95% CI)	*P* Value	aOR (95% CI)	*P* Value
Any complication	502 (38.6%)	3,781 (36.6%)	1.1 (1.0–1.2)	0.150	1.2 (1.0–1.4)	**0.010**
Infectious complication	138 (10.6%)	1,152 (11.2%)	0.9 (0.8–1.1)	0.568	1.0 (0.8–1.2)	0.683
Inpatient mortality	103 (7.9%)	431 (4.2%)	2.0 (1.6–2.5)	** < 0.001**	2.7 (2.0–3.6)	**< 0.001**
AKI	15 (1.2%)	191 (1.8%)	0.6 (0.4–1.1)	0.077	0.6 (0.4–1.1)	0.083
Cardiac complication	76 (5.9%)	331 (3.2%)	1.9 (1.5–2.4)	** < 0.001**	2.1 (1.6–2.7)	**< 0.001**
Pulmonary complication	45 (3.5%)	259 (2.5%)	1.3 (1.0–1.6)	0.059	1.4 (1.1–1.8)	**0.009**
VTE	115 (8.9%)	973 (9.4%)	0.9 (0.8-–1.1)	0.509	0.9 (0.7–1.1)	0.348

Patients aged 18–64 years	Underweight *N* = 1688	Normal weight *N* = 23,074	Bivariable logistic regression		Multivariable logistic regression	
Complication	*N* (%)	*N* (%)	OR (95%-CI)	*P* Value	aOR (95%-CI)	*P* Value
Any complication	657 (38.9%)	8,037 (34.8%)	1.2 (1.1–1.3)	**0.001**	1.2 (1.1–1.4)	**0.006**
Infectious complication	222 (13.2%)	2,583 (11.2%)	1.2 (1.0–1.4)	**0.014**	1.1 (1.0–1.3)	0.155
Inpatient mortality	69 (4.1%)	567 (2.5%)	1.7 (1.3–2.2)	**< 0.001**	1.5 (1.1–2.2)	**0.026**
AKI	33 (2.0%)	372 (1.6%)	1.2 (0.8–1.7)	0.285	1.2 (0.9–1.8)	0.244
Cardiac complication	58 (3.4%)	671 (2.9%)	1.2 (0.9–1.6)	0.216	1.1 (0.9–1.5)	0.348
Pulmonary complication	52 (3.1%)	533 (2.3%)	1.18 (1.0–1.4)	0.087	1.2 (0.9–1.4)	0.161
VTE	143 (8.5%)	2,079 (9.0%)	0.9 (0.8–1.1)	0.455	0.9 (0.8–1.1)	0.430

*aOR* adjusted odds ratio, *AKI* acute kidney injury, *OR* odds ratio, *VTE* venous thromboembolism

Bold text indicates statistical significance of *p* value less than 0.05

### Demographics, injury characteristics, and treatments in non-older patients

Within the 18–64-year-old cohort, underweight and normal weight patients had similar mean ages (37.8 vs 37.6 years, *p* = 0.519); however, underweight patients were less commonly male (55.7% vs. 68.9%, *p* < 0.001), and significant between-group differences in racial distribution were observed (Table 1). Significantly, higher rates of comorbid conditions were determined in underweight patients for five of the 18 conditions evaluated (absolute percent difference range = 0.1% to 2.7%; Table 1). On admission, injury characteristics were largely similar between the cohorts, however with higher rates of unstable pelvic ring injuries in the underweight cohort (17.3% vs. 15.4%, *p* = 0.040; Table 1). Both cohorts met ISS criteria for polytrauma (ISS > 15) on average and commonly had one column acetabular fractures (Table 1). Underweight patients were more commonly admitted to small, non-level I, non-teaching hospitals when compared to normal weight patients (Supplementary Table S3). A total of 6,321 patients were treated operatively, with non-operative treatment being employed in underweight patients more commonly than normal weight patients (79.3% vs. 74.1%, *p* < 0.001; Table 1). Of those who underwent operative fixation of their acetabular fracture, underweight patients more commonly experienced delays greater than three days than normal weight patients (37.2% vs. 32.6%, *p* < 0.001; Table 1). No significant differences in length of hospital stay were observed (10.0 days (95%-CI = 9.9–10.2) vs. 10.1 days (95%-CI = 9.5–10.7), *p* = 0.847); however, underweight patients more frequently required intubation than normal weight patients (22.6% vs. 20.6%, *p* = 0.042; Table 1).

### Outcomes in non-older patients

Significantly higher rates of any complication (38.9% vs. 34.8%, *p* = 0.001), infectious complications (13.2% vs. 11.2%, *p* = 0.014), and inpatient mortality (4.1% vs. 2.5%, *p* < 0.001) were observed in the underweight cohort (Table 2). On multivariable regression, underweight status was associated with 1.2 times greater adjusted odds of any complication (95%-CI = 1.1–1.4, *p* = 0.006) and 1.5 times greater adjusted odds of inpatient mortality (95%-CI = 1.1–2.2, *p* = 0.026). There were no other significant differences in odds of adverse outcomes, including infectious complications, AKI, cardiac complications, pulmonary complications, and VTE, after adjusting for confounding variables (all *p* > 0.05) (Table 2).

## Discussion

In a cohort of 36,391 acetabular fractures, we describe the risk of adverse outcomes conferred by underweight status in both older and younger adults. While underweight status has been previously associated with increased risk of mortality after hip fractures in older adults, there is limited data on the risk of complications after acetabular fracture, particularly within younger and middle-aged patients [8, 9]. After adjusting for confounding factors, we observed significantly greater odds of any inpatient complication and mortality in underweight patients, independent of age. Additionally, underweight older patients were at increased risk for cardiac and pulmonary complications. To our knowledge, we describe a novel independent association between underweight status and inpatient morbidity and mortality after acetabular fracture in adult patients.

The association between underweight status and risk of mortality after fractures and orthopaedic procedures within older populations has been previously described [8–10]. In two recent systematic reviews and meta-analyses by Li et al. and Yang et al., underweight BMI (< 18.5 kg/m^2^) was associated with 1.4–1.5-times greater risk of early mortality when compared to normal weight patients [8, 9]. Sarcopenia, or age-related progressive loss of muscle mass and strength, is frequently comorbid with underweight status and reflects impaired functional reserve and undernutrition [14]. In a cohort of 146 older adults with acetabular fractures, Mitchell et al. found a 4-times greater odds of one-year mortality (32.4% vs. 11.0%) among sarcopenic patients [15]. Similarly, Deren et al. demonstrated a significantly higher rate of one-year mortality (28.6% vs. 12.3%) after acetabular fracture in among 42 sarcopenic patients[16]. In our cohort of 1299 underweight older patients, we found a 7.9% inpatient mortality rate, which reflected 2.7-times greater adjusted odds of mortality when compared to the normal weight cohort. While lower than one-year mortality previously reported, this rate is still higher than the cumulative in-hospital mortality rates of 1.3–5.7% after acetabular fracture previously reported [17–19]. Additionally, here, we show an increased risk of cardiovascular and pulmonary complications in addition to greater mortality risk, thus suggesting an association between low body weight and risk of adverse cardiopulmonary events in these patients as observed with other medical conditions requiring hospitalization [10, 19–21]. Generally, low fat mass and sarcopenia portend a lower overall physiological reserve; thus, these patients may be unable to meet the metabolic demands of recovery after trauma and acetabular fracture surgery [15, 22]. To our knowledge, this study is the first to describe the increased risk of cardiopulmonary complications in elderly patients with acetabular fractures in addition to the increased risk of inpatient mortality.

Few studies have assessed the risks associated with underweight status in younger adults. Older and younger adults with acetabular fracture typically reflect distinct populations with differing injury severity, comorbidity burden, and injury mechanism. Nevertheless, we found that underweight status is associated with mortality rate after acetabular fracture independent of patient age. This finding is consistent with mortality risk after traumatic injury more broadly. In a retrospective review of 5766 polytraumatized adults, Hoffmann et al. identified underweight status as being significantly associated with in-hospital mortality on multivariable regression [23]. Similarly, in an analysis of 640 underweight adult trauma patients with an average ISS of 10, Hsieh et al. observed an increased risk of mortality in their underweight cohort [24]. However, after propensity score matching the 79 patients who died during admission to those who did not, no differences in rates of underweight status were identified. Conversely, Treto et al. determined that underweight patients who were admitted for blunt trauma with an ISS < 16 were at increased risk for inpatient mortality (5.6%) in comparison with ideal weight patients (1.8%). The potential mechanism of this increased risk was explored by Hwabejire et al. who observed that underweight patients with hemorrhagic shock had higher lactate levels and a four times higher risk of death when compared to normal weight patients. [25] To our knowledge, a single study by Waseem et al. has described the risk of mortality after acetabular fracture in adult patients. In their retrospective cohort study of 569 adult patients with either acetabular or pelvic fractures, significantly, higher inpatient (14.3% vs. 4.5%) and six-month (14.3% vs. 5.6%) mortality risk was found in the underweight cohort[11]. Supporting these prior results, our data specifically highlights that non-older underweight patients with acetabular fractures experience significantly higher rates of inpatient mortality than those with normal weight, not just older adults. Additionally, the risk of cumulative complications was higher in this group after adjusting for confounding.

Our study is not without limitations. The use of a large database carries inherent risk for classification bias related to data entry and processing, as well as limitations in follow-up and generalizability. Specific causes of death and radiographic images were not available in this dataset. As data are limited to the initial inpatient encounter, we are unable to comment on risks of mortality and post-injury sequelae after discharge. Nevertheless, the risks of complications and death during the admission after acetabular fracture are clearly substantial and remain a clinically relevant topic. Our large sample and use of multivariable analysis to address potential confounding improve our confidence in our estimates of risk. We must also note the inherent limitations in the use of BMI as a marker for nutritional status and overall health, with evidence that waist-to-hip ratios or radiographic measures may be more accurate prognostic tools [26, 27]. However, these measures have yet to be broadly adopted, while BMI is commonly assessed on admission. As such, this association remains a valid tool for risk stratifying admitted patients.

In conclusion, underweight patients with acetabular fracture are at increased risk for inpatient complications and mortality, regardless of age. These data inform the counseling of patients and caregivers about short term risks to life and health after acetabular fracture. Future studies are needed to determine whether early multidisciplinary nutritional and medical optimization can improve the poor outcomes experienced by underweight patients with acetabular fracture.

## Supplementary Information

Below is the link to the electronic supplementary material.

Supplementary file1 (DOCX 19 KB)

## Data Availability

Data analyzed in the present study are available online through the Trauma Quality Improvement Program at https://www.facs.org/quality-programs/trauma/quality/trauma-quality-improvement-program/.
