# Supplementary material for: Underweight patients experience higher inpatient complication and mortality rates following acetabular fracture
Source: Eur J Orthop Surg Traumatol. 2023 Sep 29;34(7):3543–51. doi: 10.1007/s00590-023-03739-z (PMC11490421; doi:10.1007/s00590-023-03739-z)
Supplement: Supplementary file 1 — Supplementary file1 (DOCX 19 KB) [file 590_2023_3739_MOESM1_ESM.docx]

Supplemental Tables

Supplementary Table S1. 2005 Abbreviated Injury Scale and International Statistical Classification of Diseases and Related Health Problems 10^th^ coding.

| 2005 AIS acetabular fracture | "8562..” |
| --- | --- |
| One Column | "856251" "856252" |
| Transverse | "856261" "856262" |
| Associated Both Column | "856271" "856272" |
| 2005 AIS pelvis injury | "8561.." |
| 2005 AIS femur fracture | “8530..” “8531..” “8532..” “8533..” |
| 2005 AIS tibia fracture | “8540..” “8541..” “8542..” “8543..” |
| ICD10-PCS acetabular ORIF | “0QS404Z” “0QS504Z” “0QH404Z” “0QH504Z” “0QQ40ZZ” “0QQ50ZZ” |
| ICD10-PCS acetabular CRIF | “0QS434Z” “0QS444Z” “0QS534Z” “0QS544Z” “0QH434Z” “0QH444Z” “0QH534Z” “0QH544Z” “0QQ43ZZ” “0QQ44ZZ” “0QQ53ZZ” “0QQ54ZZ” |
| Abbreviations: AIS=Abbreviated Injury Scale, CRIF=Closed Reduction Internal Fixation, ICD10=International Statistical Classification of Diseases and Related Health Problems 10th coding, ORIF=Open Reduction Internal Fixation, PCS=Procedural Coding System | |

Supplementary Table S2. Comprehensive list of covariates tested during model selection.

| All Tested Covariates | Age, sex, body mass index, all race/ethnicity covariates (American Indian, Asian, Black, Hispanic, White), all comorbid conditions (alcoholism, angina, anticoagulant use, bleeding disorders, congestive heart failure, chronic obstruction pulmonary disease, dementia, diabetes, end stage renal disease, functionally dependent health status, history of myocardial infarction, history of stroke, hypertension, mental disorder, peripheral artery disease, smoking, steroid use, and substance abuse), vitals on admission (pulse rate, respiratory rate, oxygen saturation, temperature, systolic blood pressure (SBP)), lowest SBP, Glasgow Coma Score, Injury Severity Score, AIS anatomic region severity scores 1-8, acetabular fracture type (one column, transverse, associated both column, or open fracture), associated pelvic ring injury, concomitant lower extremity injury (femoral fracture, tibial fracture), minutes spent in the emergency department before admission, admitting facility trauma level (I-III),  teaching status, total bed number (≤200, 201-400, 401-600, ≥601), not-for-profit status, interfacility transfer to treating hospital, method of definitive operative fixation of acetabular fracture (nonoperative, closed reduction internal fixation (CRIF), open reduction internal fixation (ORIF)), time to acetabular fixation, treatment by exploratory laparotomy of the peritoneal cavity, pelvic angioembolization, volume of packed red blood cells, plasma, platelets, and cryoprecipitate given during the first 4 and 24 hours of admission, and venous thromboembolism (VTE) prophylaxis type (direct thrombin inhibitor, factor Xa inhibitor, heparin, low molecular weight heparin (LMHW), warfarin, other, none). |
| --- | --- |

Supplementary Table S3. Admitting Hospital Characteristics

| **Patients Aged ≥65 Years** | **Underweight**  **N= 1,299** (%) | **Normal weight**  **N= 10,330** (%) | **P-value** |
| --- | --- | --- | --- |
| Trauma Level |  |  |  |
| I | 508 (39.1%) | 4,592 (44.5%) | **<0.001** |
| II | 459 (35.3%) | 3,277 (31.7%) |  |
| III | 113 (8.7%) | 614 (5.9%) |  |
| N/A | 219 (16.9%) | 1,847 (17.9%) |  |
| Hospital Bed Size |  |  |  |
| <200 | 107 (8.2%) | 727 (7.0%) | 0.082 |
| 200-400 | 394 (30.3%) | 3,111 (30.1%) |  |
| 401-600 | 371 (28.6%) | 2,775 (26.9%) |  |
| >600 | 427 (32.9%) | 3,717 (36.0%) |  |
| Teaching Status | 478 (36.8%) | 4,144 (40.1%) | **0.021** |
| **Patients Aged 18-64 Years** | **Underweight  N= 1,688 (%)** | **Normal weight  N= 23,074 (%)** | **P-value** |
| Trauma Level |  |  |  |
| I | 837 (49.6%) | 12,161 (52.7%) | **<0.001** |
| II | 438 (25.9%) | 6,074 (26.3%) |  |
| III | 107 (6.3%) | 534 (2.3%) |  |
| N/A | 306 (18.1%) | 4,305 (18.7%) |  |
| Hospital Bed Size |  |  |  |
| <200 | 650 (38.5%) | 1,433 (6.2%) | **<0.001** |
| 200-400 | 177 (10.5%) | 5,776 (25.0%) |  |
| 401-600 | 391 (23.2%) | 6,535 (28.3%) |  |
| >600 | 470 (27.8%) | 9,330 (40.4%) |  |
| Teaching Status | 836 (49.5%) | 12,243 (53.1%) | **0.005** |
